# Bone Health or Performance? Adaptation Response of Genetically Divergent Chicken Layer Lines to a Nutritive Calcium Depletion

**DOI:** 10.3390/ani10091645

**Published:** 2020-09-14

**Authors:** Simon Jansen, Mara Bues, Ulrich Baulain, Christin Habig, Ingrid Halle, Stefanie Petow, Ahmad Reza Sharifi, Annett Weigend, Mirja Rosmarie Wilkens, Steffen Weigend

**Affiliations:** 1 Institute of Farm Animal Genetics, Friedrich-Loeffler-Institut, 31535 Neustadt, Germany; ulrich.baulain@fli.de (U.B.); christin.habig@fli.de (C.H.); annett.weigend@fli.de (A.W.); steffen.weigend@fli.de (S.W.); 2 Institute of Physiology and Cell Biology, University of Veterinary Medicine Hannover, Foundation, 30173 Hannover, Germany; mara.bues@region-hannover.de (M.B.); mirja.wilkens@tiho-hannover.de (M.R.W.); 3 Institute of Animal Nutrition, Friedrich-Loeffler-Institut, 38116 Braunschweig, Germany; ingrid.halle@fli.de; 4 Institute of Animal Welfare and Animal Husbandry, Friedrich-Loeffler-Institut, 29223 Celle, Germany; stefanie.petow@fli.de; 5 Animal Breeding and Genetics Group, Department of Animal Sciences, University of Göttingen, 37075 Göttingen, Germany; rsharif@uni-goettingen.de; 6 Center for Integrated Breeding Research, University of Göttingen, 37075 Göttingen, Germany

**Keywords:** bone strength, egg production, eggshell quality, calcium depletion, laying hens, phylogeny, recovery

## Abstract

**Simple Summary:**

Selection for high egg production in laying hens has led to an increased calcium requirement and consequently to an intensified calcium mobilization from the bones. However, excessive demineralization can lead to osteoporosis, which is manifested by a high incidence of bone-damaged hens. In this study, we characterized the adaptation response of laying hens to a repeated dietary calcium restriction (1.09% instead of 4.26% calcium) by means of egg production, eggshell quality, body weight and bone stability. The animal model included four layer lines differing in performance level (high vs. moderately performing lines) and phylogenetic origin (white-egg vs. brown-egg layers). We assumed that the high performing lines would respond by maintaining egg production level at the expense of eggshell quality and bone health. Egg production and eggshell quality declined considerably and bone demineralization occurred in all lines. Contrary to our hypothesis, there was evidence that phylogeny rather than performance level influenced the hens′ response. The brown-egg lines appeared to be more tolerant to the calcium depletion, while the white-egg lines were more sensitive. Our findings demonstrate the influence of genetics on the adaptive capacity of chickens and underline the importance of preserving genetic variability to cope with potential future environmental challenges.

**Abstract:**

In modern laying hybrids, calcium (Ca) homeostasis is immensely challenged by daily eggshell calcification. However, excessive mobilization of Ca from bones may lead to osteoporosis, which then manifests in a high incidence of poor bone quality. The aim of this study was to characterize the hens’ adaptation response to an alternating dietary Ca restriction. The animal model consisted of four purebred layer lines, differing in laying performance (high vs. moderately performing lines) and phylogenetic origin (white- vs. brown-egg lines). According to the resource allocation theory, hens selected for high egg production were assumed to show a different response pattern to cope with this nutritive challenge compared to moderately performing lines. Data collected included egg number, egg quality traits, body weight and bone characteristics. The Ca depletion led to a temporary drop in egg production and shell quality and a loss of bone stability due to Ca mobilization. The white-egg lines response was more pronounced, whereas the brown-egg lines were less sensitive towards reduced Ca supply. Our study shows that the hens’ responsiveness to coping with a nutritive Ca depletion is not ultimately linked to genetic selection for increased egg production but rather to phylogenetic origin.

## 1. Introduction

While various functional traits have now been introduced into the selection index of laying hens, the main focus of the breeding companies remains the number of saleable eggs [1]. In female birds, there is a competitive situation, as calcium (Ca) is needed for eggshell formation and maintaining bone stability. Since up to three grams of Ca are required for each eggshell calcification process [2], modern laying hybrids′ demand for Ca is particularly high and their Ca homeostasis is stressed continually [3]. The required Ca is provided by increased intestinal absorption and stimulated bone resorption in an approximate relation of 1:0.6 [4,5]. With the onset of sexual maturity, the formation of medullary bone, which serves as a labile Ca source that can be replenished quickly [6], develops under the influence of estradiol-17β. At the same time, there is a decrease of cancellous bone volume [7,8]. This enables laying hens to meet the temporarily high demand of Ca during the periods of eggshell formation by elevated mobilisation from the bones [9]. However, if this process exceeds physiological dimensions, continuous demineralisation leads to progressive loss of not only medullary but also structural bone tissue resulting in osteoporotic bones susceptible to fractures [9,10]. High incidences of bone damage have been demonstrated [11,12] indicating major animal welfare problems and economic losses [10,13,14].

It has been reported that selection for high production efficiency in livestock species might be associated with undesirable side effects such as deficiencies in physiological and functional traits [15], due to an imbalance in resource allocation [16]. Accordingly, chicken layer lines selected for high egg production might have a reduced capacity to compensate for unfavourable physiological conditions, e.g. limited mineral resources, compared to moderately performing genotypes [17,18,19]. Differences in adaptation responses can also be attributed to phylogenetic origin, as brown- and white-egg layer lines evolved separately over a long period of time and underwent genomic changes, which may have had effects on genetic characteristics even before directional (artificial) selection for high performance began [20,21].

In the current study, we investigated the effect of a repeated dietary Ca restriction on performance traits and bone stability in four genetically divergent chicken layer lines. To address the potential implications of both the performance level and the phylogenetic origin, the animal model consisted of two brown-egg and two white-egg chicken layer lines. Within each phylogenetic group, the two lines differed in terms of egg-laying performance [22,23,24]. By characterizing the lines’ adaptation responses to dietary Ca restriction, we aimed to test line-specific responses and assess whether genetic lines reacted differently, depending on phylogenetic origin or performance level, or both. In addition, repeated Ca reductions allowed us to study if the lines’ responses were temporary and recovery occurred after supplementing feed with Ca again, or if changes were rather permanent.

We assumed that the long-term selected breeding lines for high egg production would respond by retaining their laying performance at the expense of eggshell quality and bone stability. In contrast, the moderately performing lines were supposed to respond with a decrease in performance in favour of bone health.

## 2. Materials and Methods

### 2.1. Ethical Note

The experiment was performed in accordance with German Animal Welfare Law and approved by the Lower Saxony State Office for Consumer Protection and Food Safety (LAVES) (33.19-42502-04-15/1988).

### 2.2. Animals and Housing

The experiment included four purebred chicken layer lines (*Gallus gallus domesticus*) differing in terms of egg production performance and phylogenetic origin, two white-egg layers and two brown-egg layers. The high performing lines WLA and BLA originate from a commercial breeding program (Lohmann Tierzucht GmbH, Cuxhaven, Germany) and achieve an annual egg production of about 316 eggs [22]. The moderately performing lines R11 and L68 are maintained as resource populations without any selection at the Institute of Farm Animal Genetics of the Friedrich-Loeffler-Institut (Neustadt, Germany) and achieve a laying performance of 226 (R11) and 216 (L68) eggs per year. Besides performance divergence, the animal model included a phylogenetic dimension, as the two white-egg lines (WLA and R11) are both of White Leghorn type and are closely related, but distinct from the brown-egg ones (BLA and L68), which originated from Rhode Island Red and New Hampshire breeds, respectively. The latter breed was derived from the Rhode Island Red, explaining the close phylogenetic relationship of both [21].

All chicks were hatched on the same day. The chicks were tagged with wing bands at hatch for identification and sorted by sex. They were reared in a floor system under standard conditions. Information on the light program is given in the Supplementary Table S1. From the beginning of the 24th week of age, the birds were exposed to a light-dark cycle of 14 h L: 10 h D. Customary complete feeds for chicks (until 6 weeks of age; 11.8 MJ AME_N_/kg dry matter (DM), 210.0 g/kg crude protein, 40.0 g/kg crude fat, 35.0 g/kg crude fiber, 60.0 g/kg crude ash, 9.5 g/kg Ca, 6.5 g/kg phosphorous) and pullets (from 7 to 17 weeks of age; 11.4 MJ AME_N_/kg DM, 155.0 g/kg crude protein, 40.0 g/kg crude fat, 45.0 g/kg crude fiber, 50.0 g/kg crude ash, 8.5 g/kg Ca, 5.5 g/kg phosphorous) were offered *ad libitum*. At 16 weeks of age, 132 pullets (33 birds per layer line) were transferred to six 8 m² floor pens each littered with wood-shavings and equipped with nipple drinkers, two feeding troughs and four nest boxes. Each pen was occupied in equal proportions with 22 randomly chosen hens of WLA/L68 or BLA/R11 combination. The lines were combined to meet the limited number of nest boxes in the pens, as brown-egg lines use the nests earlier during the day than white-egg lines [25]. Furthermore, combining white-egg and brown-egg lines enabled a separate recording of the egg data for each chicken line even when kept together in the same floor pen. In this way, four Ca restricted pens and two control pens were formed resulting in two temporarily Ca deficient (DEF) and one control group (CON) per chicken layer line.

### 2.3. Experimental Procedure

**Figure 1:**
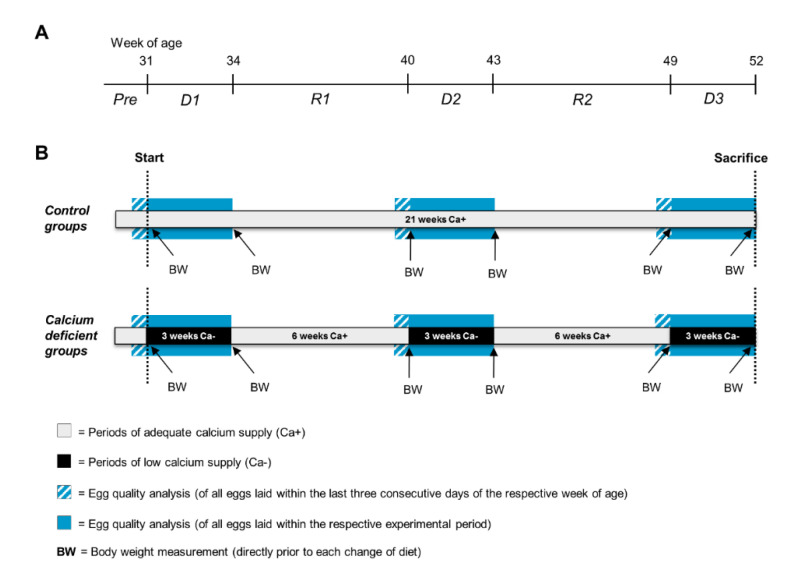
Schematic illustration of the experimental periods and related procedures. (**A**) Age of the animals in weeks and experimental periods (Pre: preliminary period; D1: first calcium depletion period; R1: first recovery period; D2: second calcium depletion period; R2: second recovery period; D3: third calcium depletion period). (**B**) Dietary calcium supply and experimental procedures. Control groups were fed with adequate calcium diet (Ca+) continuously. Calcium deficient groups were fed alternatingly with low calcium diet (Ca−) (black sections) and adequate Ca+ feed. Egg quality analysis was performed on all eggs laid within the last three consecutive days of periods Pre, R1 and R2 (blue striped sections), as well as during the entire phases of Ca− (blue sections). Body weighing (BW) was performed directly prior to each change of diet.

The experiment lasted from the beginning of the 31st to the end of the 51st week of age. Two customary wheat-soya-based diets for layers were fed *ad libitum*, which only varied in terms of Ca content. The ingredients and chemical composition of the layer diets are listed in Supplementary Table S2. With regard to nutritional recommendations for high performing laying hens [26], the diets’ Ca content can be classified as deficient (Ca−, 1.09%) and adequate (Ca+, 4.26%). The Ca+ diet was fed to all hens from 18 to 30 weeks of age. While the Ca+ diet was fed to the control groups continuously during the whole experiment, the Ca deficient groups were provided with both the Ca+ and the Ca− diet, alternatingly. In the latter case, a 21-day period of Ca depletion (Ca−) was followed by a 44-day recovery phase (Ca+) twice, followed by a third period of Ca restriction (Ca−). This resulted in a preliminary period (Pre, week 18–30), three periods of Ca depletion (D1, week 31–33; D2, week 40–42; D3, week 49–51) with two intermediate recovery periods (R1, week 34–39; R2, week 43–48). Figure 1 gives an overview on the experimental periods and the related procedures.

Data collection included traits on production performance and bone stability. Pen-level egg production, including eggshell breakages and defects, was recorded daily. The total laying rate and rate of broken eggs were calculated on a daily basis by dividing the number of eggs by the number of hens. Feed consumption (g) was recorded weekly on pen-level as the difference between the feed weighed in the feeding trough and the refusals. However, due to technical obstacles, feed consumption is only available from period D2 onwards. Immediately prior to changing the diet, the body weight (g) was measured using a digital table scale (CPA 16001S, Sartorius, Göttingen, Germany). Egg quality measurements included all eggs laid during Ca depletion periods (D1, D2 and D3) as well as those eggs laid within the last three consecutive days of the preliminary period (Pre), and the two recovery periods (R1 and R2). Eggshell breaking strength (N) was determined using a testing machine that showed the maximum load that was required to break the eggshell. Egg weight (g) and eggshell weight (g) were recorded using a digital table scale (Type 3709, Sartorius, Göttingen, Germany). For the latter, the eggs were emptied with a spoon and the shells were dried for 30 s in a microwave (800 watt). Eggshell thickness (mm) was measured near the equator using a caliper with an accuracy of 0.01 mm after removing the shell membranes.

At the end of the 51st week of age, the hens were euthanized by carbon dioxide inhalation. Keel bones, tibiotarsi and humeri of the birds were extracted and the adherent tissue removed. The bones were vacuum-packed and stored frozen (−20 °C) until further examination. After measuring the bone weight (g), length (mm) and thickness (mm) of the left tibiotarsus and the left humerus, their radio density was determined given as millimetres of aluminum equivalent (mm Al eq) [27]. The diaphyseal cortical bone proportion (“cortical area”) of the left tibiotarsus cut surface was assessed planimetrically [28]. The right long bones were used for assessing bone breaking strength (N) via a three-point bending test as described by Jansen et al. [22]. At slaughter, the keel bones were visually examined for the presence of fractures, indicated by fracture lines or callus formation.

### 2.4. Statistical Analyses

Data analysis was carried out using SAS 9.4 (SAS Institute Inc. Cary, NC, USA, 2017). For each layer line, the sample size was *n* = 22 (DEF groups) and *n* = 11 (CON groups).

Both the total laying rate and the rate of broken eggs were analyzed within the layer lines, applying a univariate regression approach using the MIXED procedure, modelling the linear relationship between the laying rate or rate of broken eggs and the day of depletion according to the following model:

(1) $$\gamma_{i}=\;\beta_{0}+\beta_{1}x_{i}+\varepsilon_{i}$$

where $\gamma_{i}$ is the trait under consideration, $\beta_{0}$ is the intercept, $\beta_{1}$ is the slope, $x_{i}$ is the independent variable “day of depletion” and $\varepsilon_{i}$ is the random error variance.

Concerning egg quality traits, we first examined whether there were significant differences between the dietary groups of the layer lines in the preliminary (Pre) or recovery phases (R1, R2). For the depletion periods (D1, D2, D3), an analysis of covariance was applied in order to fit regression curves considering the time during the depletion as a covariate term up to 4 polynomial degrees and the fixed effect of dietary treatment as well as significant interactions between the dietary treatment and the covariate (day of depletion) up to degree 4 of polynomials [29]. For the analysis of the eggshell weight data, the egg weight was considered as a covariate. In a backward selection approach, the Wald F-statistics were used to determine the significance of fixed effects. Egg quality data were analyzed with the MIXED procedure of SAS. Least squares means (LSM) were estimated by applying the LSMEANS statement. Significant differences between LSM were tested using Tukey’s HSD (honestly significant difference) test. Statistical significance was set at *p* < 0.05. Standard errors of LSM were calculated as described by Littell et al. [29].

The impact of time of measurement, dietary treatment and their interaction on the body weight was analyzed within layer line using the GLIMMIX procedure according to the following model:

(2) $$\gamma_{ijkm}=\mu +T_{i}+D_{j}+T_{i}\times D_{j}+S_{k}+\varepsilon_{ijkm}$$

where $\gamma_{ijkm}$ is the trait under consideration; μ is the general mean; $T_{i}$ is the fixed effect of time of measurement (i = 1 to 6); $D_{j}$ is the fixed effect of the diet (j = 1, 2); $T_{i}\times D_{i}$ is the fixed effect of interaction between time of measurement and diet; $S_{k}$ is the random effect of sire (k = 1 to 66); and $\varepsilon_{ijkm}$ is the random error variance. Tukey’s HSD-Test was performed for multiple comparisons of means. Statistical significance was set at *p* < 0.05.

Bone analysis took place at the end of the study. At this point, the impact of layer line, dietary treatment and their interaction on the bone characteristics were analyzed using the GLIMMIX procedure according to the following model:

(3) $$\gamma_{ijkm}=\mu +LL_{i}+D_{j}+LL_{i}\times D_{j}+S_{k}+\varepsilon_{ijkm}$$

where $\gamma_{ijkm}$ is the trait under consideration; μ is the general mean; $LL_{i}$ is the fixed effect of layer line (i = 1 to 4); $D_{j}$ is the fixed effect of the diet (j = 1, 2); $LL_{i}\times D_{j}$ is the interaction between layer line and diet; $S_{k}$ is the random effect of sire (k = 1 to 66); and $\varepsilon_{ijkm}$ is the random error variance. Tukey’s HSD-Test was performed for multiple comparisons of means. Statistical significance was set at *p* < 0.05.

The results of the keel bone examination have been dichotomized, differentiating between “at least one fracture was present” (score 1) and “no fractures were present” (score 0). The effect of the dietary treatment on the fracture occurrence was analyzed by means of Fisher′s exact test separately for each layer line using the FREQ procedure.

## 3. Results

### 3.1. Laying Performance

**Figure 2:**
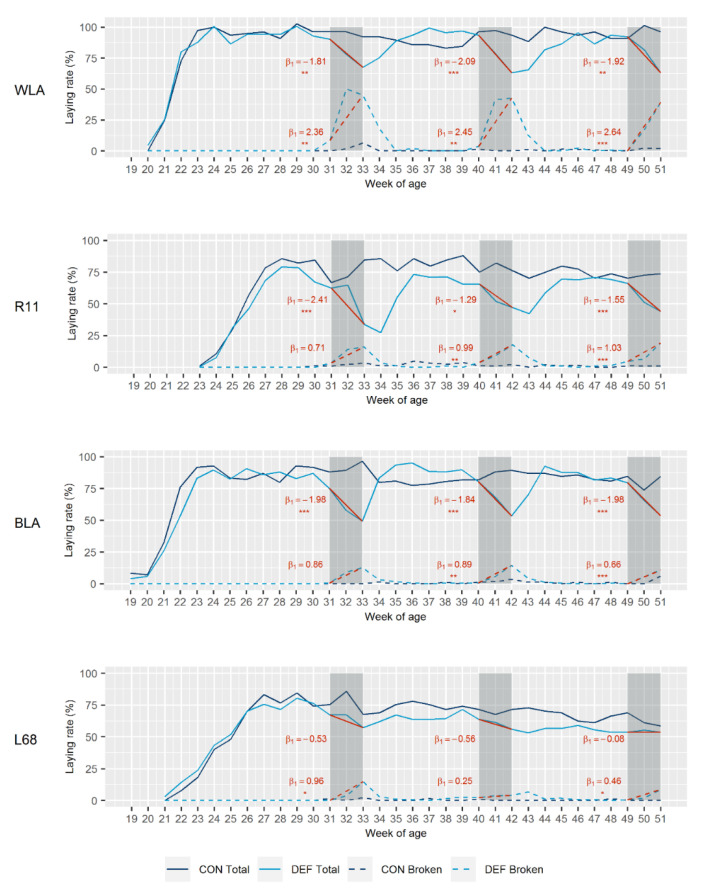
Total laying rate (solid lines) and laying rate of broken eggs (dashed lines) in control (CON) and calcium (Ca) deficient (DEF) groups of four layer lines (white-egg layers (WLA, R11), brown-egg layers (BLA, L68)). Grey-shaded sections represent the periods of Ca depletion. Linear regression curves and the corresponding coefficients (*β*_1_) between day of depletion and total laying rate or laying rate of broken eggs are given in red. Significant regression coefficients are marked with asterisks (* *p* < 0.05; ** *p* < 0.01; *** *p* < 0.001). DEF groups: *n* = 22; CON groups: *n* = 11.

Figure 2 illustrates the total laying rate of the dietary groups of the four layer lines. In addition, the linear relationship between the total laying rate and the day of the respective depletion period is indicated. At 19 (BLA) and 20 (WLA) weeks, laying maturity was reached earlier in the high performing lines than in the moderately performing ones, whose first eggs were laid at week 21 (L68) and 23 (R11). The DEF groups of WLA, R11 and BLA showed a marked decline in egg production among all depletion periods. Highly significant, negative regression coefficients (*β*_1_) between time of progressive Ca depletion and the laying rate were found in these lines, resulting in average values across the three depletion phases of *β*_1_ = −1.94 (WLA), *β*_1_ = −1.75 (R11) and *β*_1_ = −1.93 (BLA). In the case of the line WLA, for example, this means that per day of Ca depletion, the laying rate decreased by 1.94%. After reconversion to adequate Ca supply, the initial performance level was regained consistently in these lines, as the intercept (*β*_0_) of the regression curves varied only a little between the depletion periods (Figure 2). This indicates a recovery in laying performance. Since both dietary groups of the moderately performing brown-egg line L68 declined more or less equally in the course of the experimental period, there was not such a strong response to Ca depletion in this line as there was for the other lines. The corresponding regression coefficients were not significant.

Figure 2 also shows the rate of broken eggs, i.e., eggshell breakages and defects, and the linear relationship between this variable and the day of the depletion period. Increased incidences of eggshell breakages and defects were observed in the DEF groups of all lines. In this instance, the high performing white-egg line WLA showed a considerably higher incidence, as reflected by average regression coefficients of *β*_1_ = 2.48 (WLA), *β*_1_ = 0.91 (R11), *β*_1_ = 0.80 (BLA) and *β*_1_ = 0.56 (L68). In all lines, however, the rate of broken eggs declined to the initial level of below 1.5% within two weeks of the recovery phases.

### 3.2. Egg Quality

**Figure 3:**
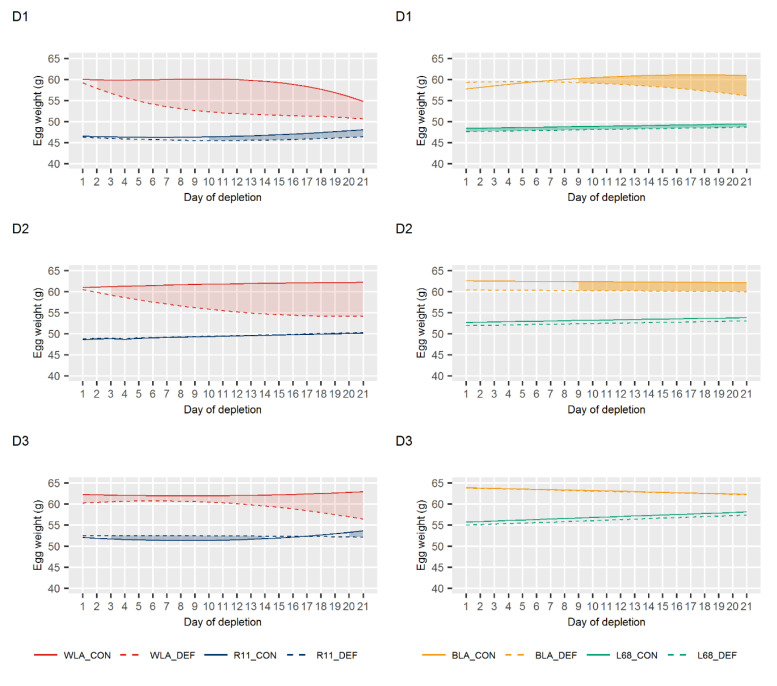
Least squares means for egg weight in control (CON) and calcium (Ca) deficient (DEF) groups of four layer lines (WLA, R11, BLA, L68) during periods of Ca depletion (D1, D2, D3). The filled in areas indicate when both dietary groups of the lines differ significantly at *p* < 0.05.

**Figure 4:**
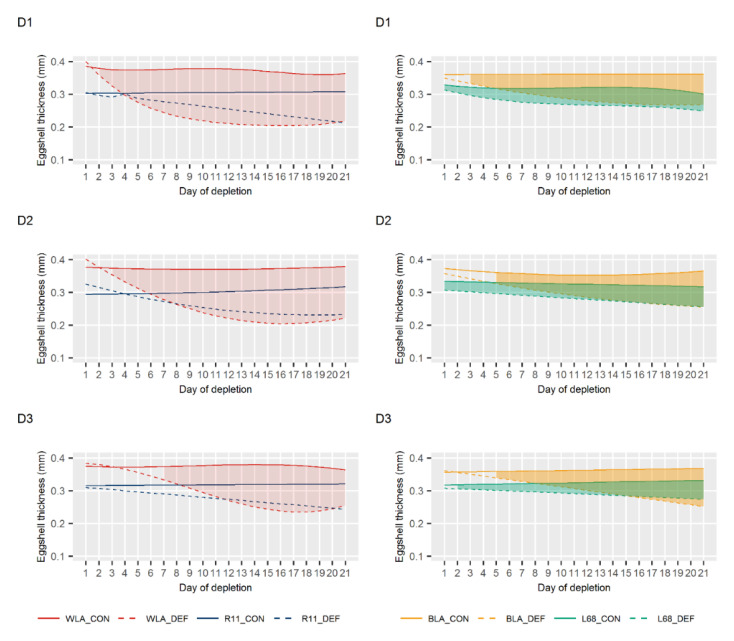
Least squares means for eggshell thickness in control (CON) and calcium (Ca) deficient (DEF) groups of four layer lines (WLA, R11, BLA, L68) during periods of Ca depletion (D1, D2, D3). The filled in areas indicate when both dietary groups of the lines differ significantly at *p* < 0.05.

**Figure 5:**
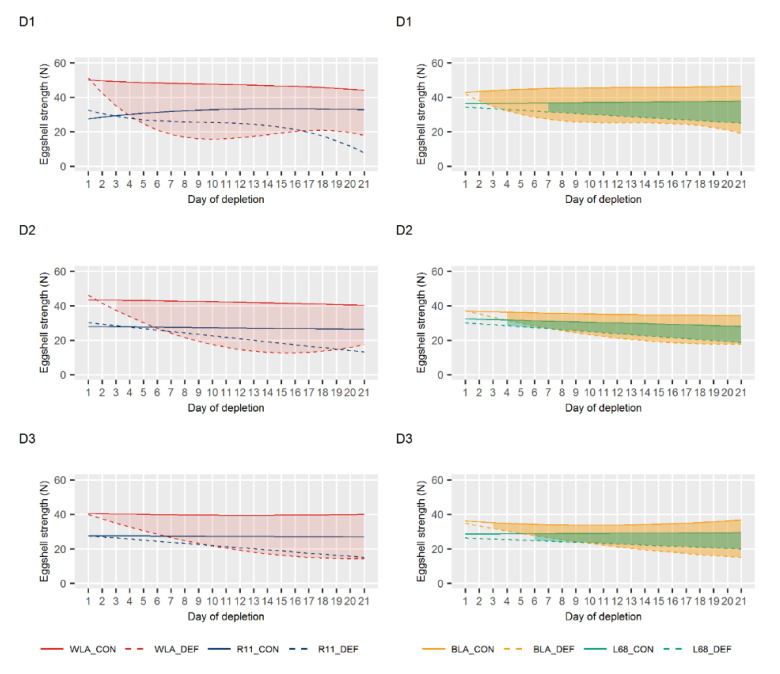
Least squares means for eggshell breaking strength in control (CON) and calcium (Ca) deficient (DEF) groups of four layer lines (WLA, R11, BLA, L68) during periods of Ca depletion (D1, D2, D3). The filled in areas indicate when both dietary groups of the lines differ significantly at *p* < 0.05.

The progression of egg quality traits during the periods of Ca depletion is shown in Figure 3 (egg weight), Figure 4 (eggshell thickness) and Figure 5 (eggshell breaking strength). Taking all periods into account, significant differences between DEF and CON groups of line WLA were observed consistently and mostly earlier than in all other layer lines. While the DEF group of line WLA responded significantly for most traits within the first three days, the dietary groups of line R11 only tended to differ in terms of eggshell breaking strength and thickness. The only significant differences found in line R11 were in egg weight in periods D1 and D3. However, these differences were rather minor. In Ca deficient BLA hens, a significant egg weight decline was observed from the ninth day on (except in D3), whereas for the other traits, a significant response occurred even within the first five days. Although Ca depletion also caused a decrease in egg quality in line L68, a more pronounced decline was observed in its high performing counterpart BLA. These observations are supported by the results on the eggshell weight shown in Supplementary Figure S1.

No significant differences in egg quality were observed between the two dietary groups within each of the layer lines WLA, R11 and BLA at the end of the periods with a sufficient Ca supply, i.e., at the end of the periods Pre, R1 and R2 (Supplementary Table S3). Thus, egg quality during periods D2 and D3 has not been affected by prior depletions, suggesting a recovery from restricted Ca supply. Only in line L68, rather small but significant differences in eggshell weight and eggshell thickness were observed in period R1. Whether this represents an aftereffect of period D1 cannot be ruled out, but it seems rather unlikely, since no differences occurred in both the Pre and R2 periods.

### 3.3. Body Weight

**Figure 6:**
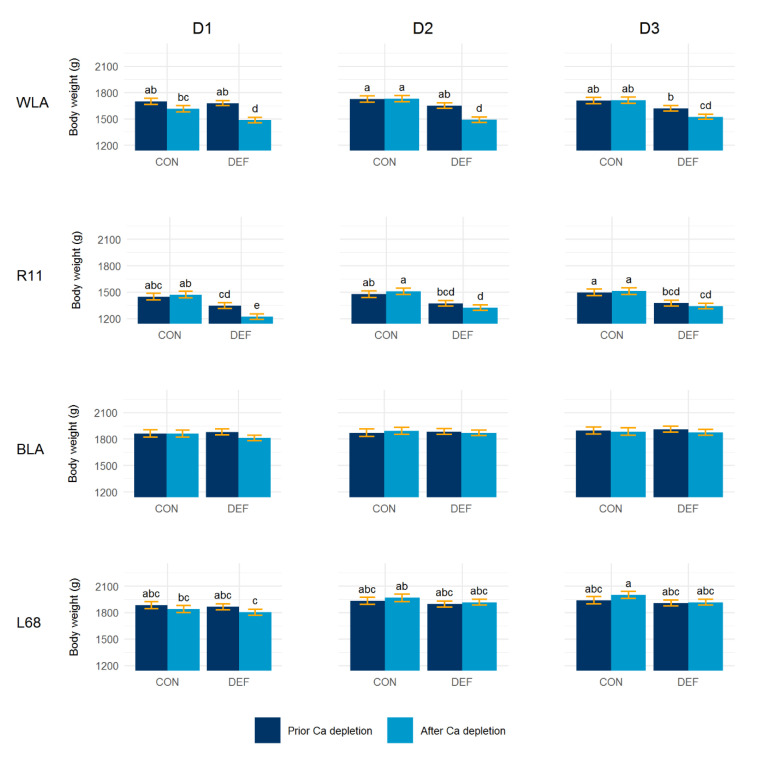
Least squares means ± standard errors for body weight prior and after calcium (Ca) depletion during the three restriction periods (D1, D2, D3), in control (CON) and Ca deficient (DEF) groups of four layer lines (WLA, R11, BLA, L68). DEF groups: *n* = 22; CON groups: *n* = 11. ^a,b,c,d^ means with different letters within layer lines differ significantly at *n* < 0.05.

Figure 6 illustrates the body weight prior and after the depletion periods according to model (2). Within layer lines and over all time points, the ANOVA revealed a significant dietary effect on the body weight (WLA: *p* < 0.001; R11: *p* < 0.0001; L68: *p* = 0.0381). In contrast, no dietary effect was found for line BLA (*p* = 0.7457). Significant body weight reduction was only observed in the DEF groups of the white-egg lines, whereas no weight changes were evident in the brown-egg lines. While the dietary groups of line R11 differed only in period D1, the DEF group of line WLA had a significant weight reduction in all depletion periods suggesting a distinct response to the Ca depletion. However, the lines fully recovered from this, as no significant differences between the body weight values at the end of the periods Pre, R1 and R2 were found.

### 3.4. Feed Consumption

The feed consumption for the WLA/L68 or BLA/R11 combination is shown in the Supplementary Table S4. Overall, the feed consumption ranged between 100 g and 120 g per animal and day. Apart from certain general fluctuations, the descriptive analysis suggested a reduced feed consumption of the DEF groups during all depletion periods, and this was reversed in the following recovery phases.

### 3.5. Bone Characteristics

**Figure 7:**
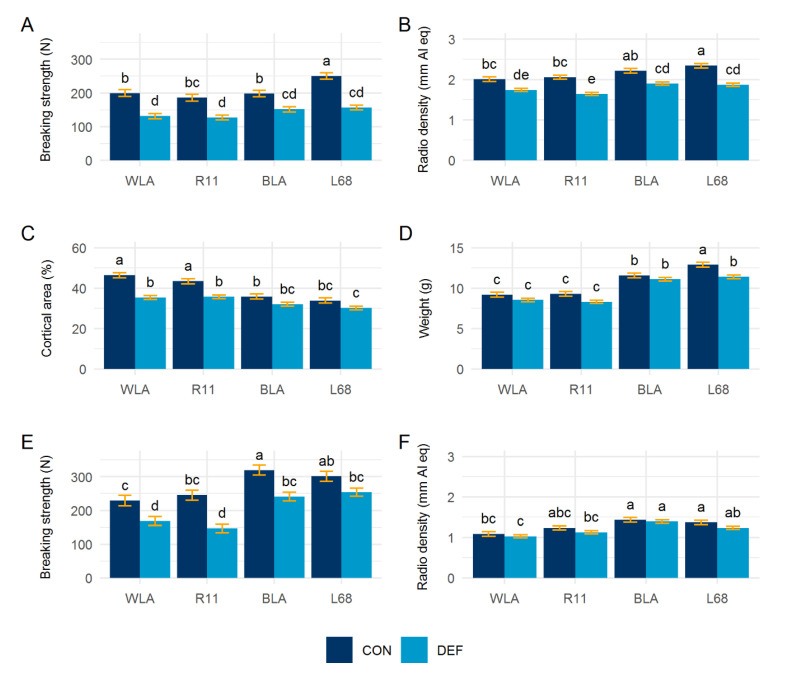
Least squares means ± standard errors for (**A**) bone breaking strength, (**B**) radio density, (**C**) cortical area and (**D**) weight of the tibiotarsus; (**E**) bone breaking strength and (**F**) radio density of the humerus in control (CON) and calcium deficient (DEF) groups of four layer lines (WLA, R11, BLA, L68). DEF groups: *n* = 22; CON groups: *n* = 11. ^a, b, c, d, e^ means with different letters differ significantly at *p* < 0.05

The LSM of examined bone characteristics are shown in Figure 7. All bone parameters were significantly influenced by the layer line (*p* < 0.0001). The breaking strength, radio density, weight and cortical area of the tibiotarsus (*p* < 0.0001) as well as the breaking strength (*p* < 0.0001) and radio density (*p* = 0.0193) of the humerus were significantly influenced by the dietary treatment. The layer line by diet interaction was only significant for the breaking strength (*p* = 0.0218) and cortical area (*p* = 0.0018) of the tibiotarsus. With the exception of the humerus in line L68, bone breaking strength was significantly decreased in the DEF groups of all layer lines. The comparison of means further showed that radio density was only affected in the tibiotarsus, where the DEF groups possessed significantly lower bone density. The DEF groups of the white-egg lines WLA and R11 showed significantly declined cortical area of the tibiotarsus, while for both brown layers the difference was rather small and not significant. The Ca deficit led to a slight weight reduction of the tibiotarsus in all lines, which was only significant in line L68.

Results of postmortem examination of the keel bones are shown in the Supplementary Figure S2. Only in the DEF group of line R11 was the proportion of hens with at least one fracture significantly higher than in the CON group. The dietary groups of the other lines did not differ significantly regarding the occurrence of keel bone fractures.

## 4. Discussion

Considerable response to Ca depletion was observed among the layer lines. Our results support the findings of previous studies, in which dietary Ca restriction led to marked and sudden reduction of egg production [30,31,32]. Consistent with the literature, we also found that hypocalcaemia resulted in progressive reduction of eggshell production [32,33,34]. In line with Jiang et al. [35], our study showed that decreased eggshell quality leading to increasing incidence of egg breakages and defects was most likely the result of an inadequate Ca supply.

In contrast to Gilbert and Blair [30] and Luck and Scanes [36], who fed a diet of 0.05% Ca for six weeks and 0.03% Ca for three weeks, respectively, no evidence of cessation of laying activity was observed. A possible explanation for this might be that only drastically reduced Ca contents cause a suspension, while minor depletions, like in our case, merely lead to a performance decline [30,37].

Although all lines showed a certain performance depression, there were differences between the layer lines. Here, the white-egg lines seem to have been more sensitive to the Ca depletion, as they showed not only a greater drop in egg production, but also a higher proportion of defective eggs reflecting impaired eggshell quality. Conversely, despite the same dietary stress, the brown-egg lines retained a higher proportion of intact eggs, possibly because their skeletal system was a relatively larger Ca reservoir compared to the white-egg lines. Moreover, there was evidence that within the phylogenetic groups, a greater decline in egg production and quality occurred in the high performing lines. For example, eggshell quality traits were more decreased in the high performing WLA and BLA lines than in their moderately performing counterparts.

We observed substantial recovery in all layer lines after returning to the adequate Ca supply. Our results therefore indicate that the physiological stress induced by the administration of 1.09% Ca provoked adaptation response but did not cause permanent impairment of the hens’ performance. This is in accordance with previous studies, as Summers et al. [31] found significantly heavier eggs with stabilized eggshells after changing from a previous 28-day supply of 1.50% Ca to 2.96% Ca. Immediate improvement, namely increased eggshell strength and laying performance, was also reported for repeated eight-week periods of Ca depletions [32].

The Ca deficiency led to a decrease in body weight and tended to reduce feed consumption. While a tendency of lower feed intake was observed in all DEF groups, significant body weight reduction only occurred in the white-egg lines. This may reflect phylogenetic differences. There seems to be a consensus that Ca deficiency leads to reduced feed intake [31,32,38]. In contrast, reports on body weight are inconsistent. Different studies have shown a decreasing [34], increasing [39] or even missing effect of diet on body weight [40]. Irrespective of the underlying mechanism, the body weight of the WLA and R11 hens recovered each time after reconversion to adequate Ca supply.

We observed a significant decrease in bone breaking strength and, to some extent, radio density, which is in accordance with previous studies [32,38,41]. However, our investigations revealed layer-line-specific differences. This especially applied to the white-egg lines, both of which showed a significant degradation of cortical bone tissue. While the medullary Ca reserves were sufficient to buffer temporary Ca fluctuations, structural bone, i.e., cortical and trabecular bone, was demineralized during prolonged Ca reduction [4,42]. Cortical bone resorption therefore suggests that the medullary Ca reservoir was insufficient in the WLA and R11 lines. The lines BLA and L68, on the other hand, had larger and thicker bones [22], which probably also had a higher absolute medullary content that provided sufficient Ca so that no cortical bone had to be resorbed. Therefore, the brown-egg lines probably had a higher capacity to tolerate Ca depletion thus reflecting a phylogenetic component. That the bone breaking strength was equally impaired in both phylogenetic groups emphasized that medullary bone contributed to overall fracture resistance [8,43].

Taken together, Ca depletion caused both decreased eggshell production and increased bone demineralization. Given the ongoing debate about mismatched resource allocation, according to which, egg production is prioritized [19,44], our results may reflect the hens’ attempt to maintain reproductive performance at the expense of bone stability. Contrary to our assumptions, this was the case for all layer lines. However, the response to the Ca deficit was differently pronounced, which possibly represented the line-specific adaptation potential of the layer lines examined here. For this, lines WLA and L68 responded most differently, as the Ca restriction had the most striking effects on WLA hens. On the other hand, only minor effects were observed for line L68, but this may have been caused by a lower Ca demand in general. Despite this line-specificity, there is evidence that the hens responded differently depending on their phylogenetic origin.

While the present study phenotypically focuses mainly on egg production traits and bone characteristics, further studies at the molecular level may help to characterize the adaptation response and explain the differences between the layer lines observed in the current experiment. Here, the description of blood parameters relevant for Ca homeostasis, such as ionized Ca, total blood Ca, vitamin D3 and phosphorus, and the expression level of epithelial Ca transport proteins may be used for a more comprehensive characterization of the adaptation response. In a follow-up study, the use of single cage housing instead of a floor management system would allow the administration of Ca restrictions adapted to the egg production of the individual layer lines. Moreover, cage housing would avoid the hens eating eggs, which, although not observed in the present study, could lead to a bias in the data.

## 5. Conclusions

In this study, we characterized the adaptation response of genetically divergent chicken layer lines to repeated transient periods of calcium (Ca) depletion. It could be shown that laying hens apparently compensate for a temporary lack of Ca in the characteristics studied, albeit in different ways. Contrary to our hypothesis, our results did not indicate a major influence from selection for high egg numbers on the response to Ca depletion. Although layer-line-specific responses were observed, overall, the phylogenetic origin tended to be one of the determining factors with the brown-egg lines showing a higher tolerance to the Ca deficit. This was probably due to a more favorable body constitution in which the skeletal system was able to provide a higher amount of Ca without severe health restrictions. It seems essential to maintain the hens’ adaptability to cope with changing or disadvantageous environmental conditions. This inevitably requires the preservation of genetic variation for adaptive performance.

## Supplementary Materials

The following are available online at https://www.mdpi.com/2076-2615/10/9/1645/s1, Figure S1: Least squares means for eggshell weight, Figure S2: Occurrence of keel bone fractures, Table S1: Light program, Table S2: Ingredients and nutrient composition of the layer diets, Table S3: Egg quality traits during the last three consecutive days of preliminary, first and second recovery period, Table S4: Mean daily feed consumption.
