# Supplementary material for: Bone Health or Performance? Adaptation Response of Genetically Divergent Chicken Layer Lines to a Nutritive Calcium Depletion
Source: Animals (Basel). 2020 Sep 14;10(9):1645. doi: 10.3390/ani10091645 (PMC7552123; doi:10.3390/ani10091645)
Supplement: Supplementary file 1 [file animals-10-01645-s001.zip › Supplement_FigureS1.pdf]

Supplementary Material

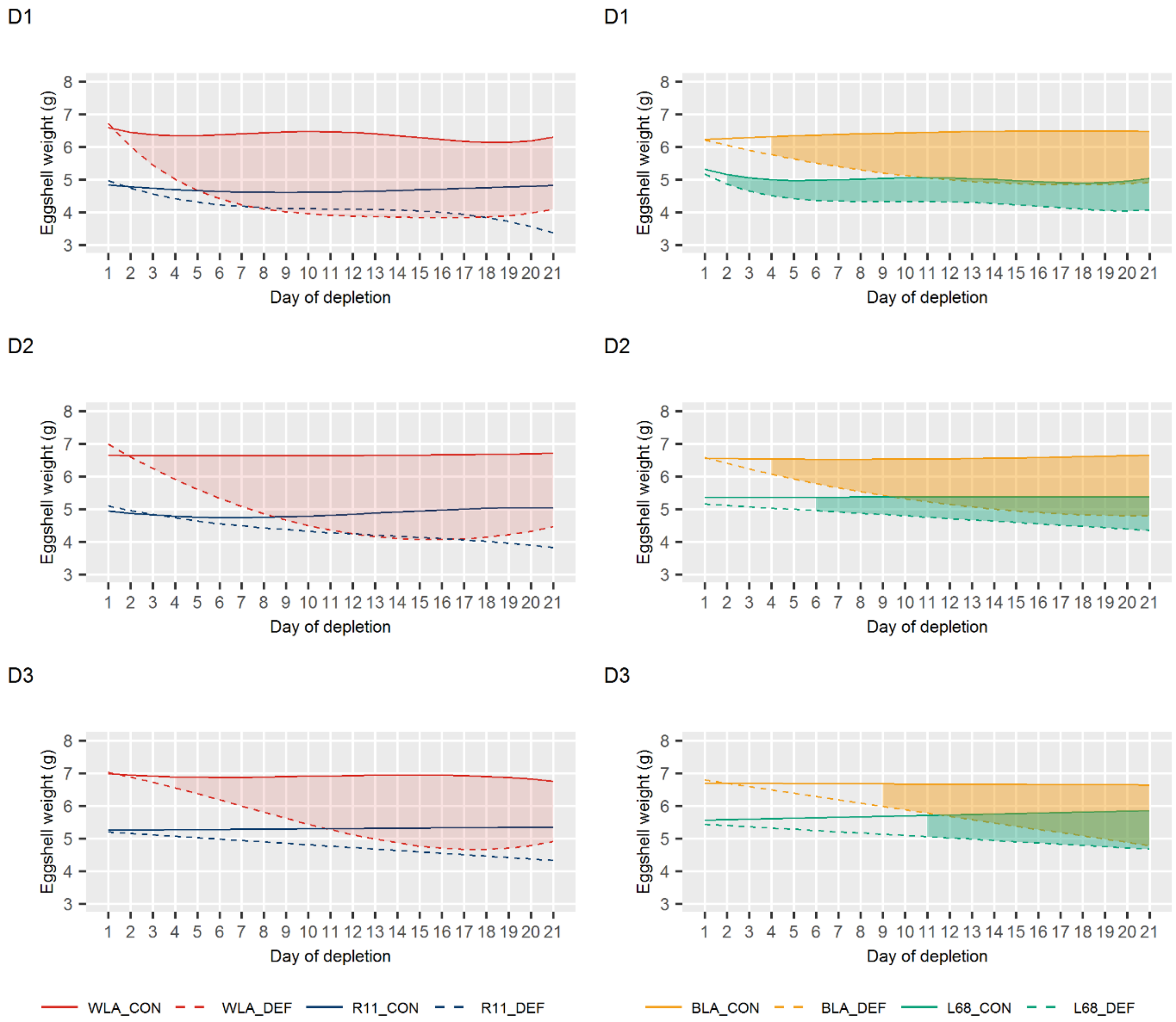

Figure S1. Least squares means for eggshell weight in control (CON) and calcium deficient (DEF) groups of four layer lines (WLA, R11, BLA, L68) during periods of calcium depletion (D1, D2, D3). The filled in areas indicate when both dietary groups of the lines differ significantly at $p < 0.05$.
