# Supplementary material for: Bone Health or Performance? Adaptation Response of Genetically Divergent Chicken Layer Lines to a Nutritive Calcium Depletion
Source: Animals (Basel). 2020 Sep 14;10(9):1645. doi: 10.3390/ani10091645 (PMC7552123; doi:10.3390/ani10091645)
Supplement: Supplementary file 1 [file animals-10-01645-s001.zip › Supplement_FigureS2.pdf]

Supplementary Material

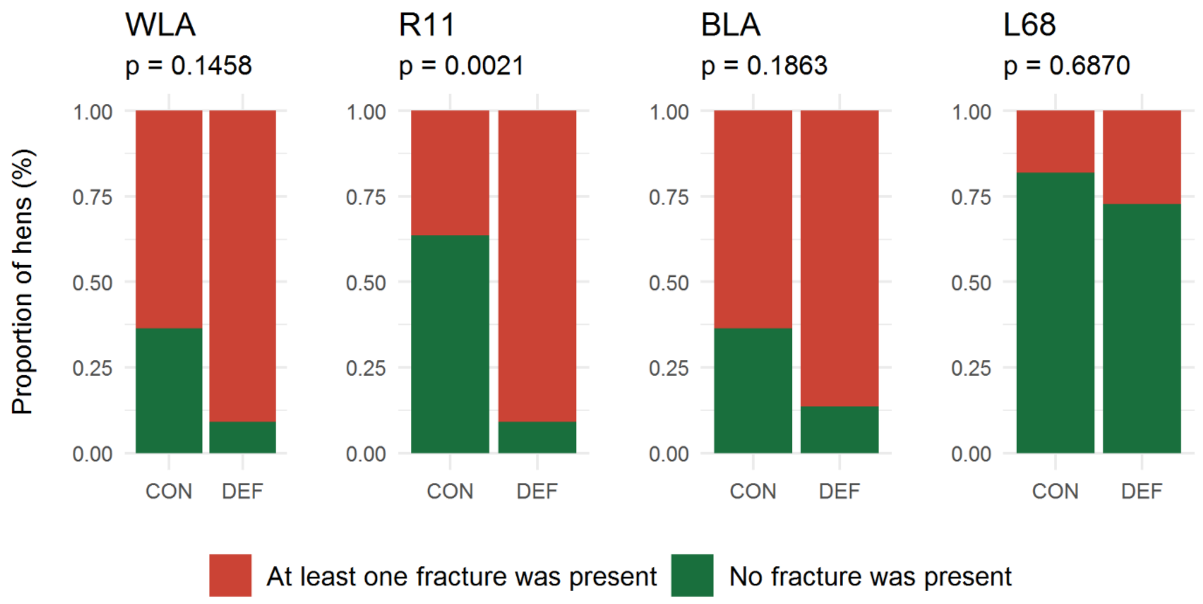

Figure S2. Occurrence of keel bone fractures in control (CON) and calcium deficient (DEF) groups of four layer lines (WLA, R11, BLA, L68). Each bar represents the total amount of examined keel bones of one layer line x diet combination (Fisher's exact test, $p < 0.05$).
