# Supplementary material for: Bone Health or Performance? Adaptation Response of Genetically Divergent Chicken Layer Lines to a Nutritive Calcium Depletion
Source: Animals (Basel). 2020 Sep 14;10(9):1645. doi: 10.3390/ani10091645 (PMC7552123; doi:10.3390/ani10091645)
Supplement: Supplementary file 1 [file animals-10-01645-s001.zip › Supplement_TableS1.pdf]

Supplementary Material

Table S1. Light program.

Week of age	Time		Day length [h]	Light intensity [Lux]
	Start	End		
Day 1	-	-	24.0	20.0
1	4:00 am	7:00 pm	15.0	20.0
2	4:30 am	7:00 pm	14.5	20.0
3	5:30 am	7:00 pm	13.5	20.0
4	6:00 am	7:00 pm	13.0	20.0
5	7:00 am	7:00 pm	12.0	20.0
6	7:00 am	6:00 pm	11.0	10.0
7	7:00 am	5:00 pm	10.0	10.0
8	7:30 am	4:30 pm	9.0	10.0
9	7:30 am	3:30 pm	8.0	10.0
10	7:30 am	3:30 pm	8.0	10.0
11	7:30 am	3:30 pm	8.0	10.0
12	7:30 am	3:30 pm	8.0	10.0
13	7:30 am	3:30 pm	8.0	10.0
14	7:30 am	3:30 pm	8.0	10.0
15	7:30 am	3:30 pm	8.0	10.0
16	7:30 am	3:30 pm	8.0	10.0
17	7:00 am	3:30 pm	8.5	10.0
18	6:30 am	3:30 pm	9.0	10.0
19	6:30 am	4:00 pm	9.5	10.0
20	5:30 am	4:00 pm	10.5	10.0
21	4:30 am	4:00 pm	11.5	10.0
22	4:00 am	4:30 pm	12.5	10.0
23	3:30 am	5:00 pm	13.5	10.0
24 - 52	3:30 am	5:30 pm	14.0	10.0
