# Supplementary material for: Bone Health or Performance? Adaptation Response of Genetically Divergent Chicken Layer Lines to a Nutritive Calcium Depletion
Source: Animals (Basel). 2020 Sep 14;10(9):1645. doi: 10.3390/ani10091645 (PMC7552123; doi:10.3390/ani10091645)
Supplement: Supplementary file 1 [file animals-10-01645-s001.zip › Supplement_TableS2.pdf]

Supplementary Material

Table S2. Ingredients and nutrient composition of the layer diets.

Diet	Adequate Calcium diet (Ca+)	Low Calcium diet (Ca-)
**Ingredients (%)**		
Wheat	39.74	36.81
Wheat bran	-	11.00
Corn	20.00	25.00
Soybean toasted	10.63	-
Soybean meal toasted	8.00	10.71
High protein soybean meal toasted	5.00	5.00
Lucerne pellets	2.44	5.58
Soybean oil	2.00	2.00
Calcium phosphate	2.46	1.79
Calcium carbonate	8.15	0.53
Sodium chloride	0.42	0.40
DL-Methionine	0.16	0.15
Lysine HCL	-	0.02
Premix [1]	1.00	1.00
**Nutrient composition (on dry matter basis)**		
Crude protein (%)[2]	17.10	16.60
Crude fat (%)[2]	6.40	5.00
Starch (%)[2]	38.20	42.70
Sucrose (%)[2]	3.30	3.40
AME_N / kg Dry matter (MJ) [3,4]	11.60	11.90
Calcium (%)[2]	4.26	1.09

[1] Premix – hens: feed additives (per kg premix): Vitamin A, 1,000,000 IU; Vitamin D3, 250,000 IU; Vitamin E, 2,000 mg; Vitamin B1, 250 mg; Vitamin B2, 700 mg; Vitamin B6, 400 mg; Vitamin B12, 2,000 µg; Vitamin K3, 400 mg; Nicotin amide, 4,000 mg; Calcium-D-pantothenate, 1,000 mg; Folic acid, 60 mg; Biotin, 2,500 µg; Choline chloride, 40,000 mg; Fe, 4,000 mg; Cu, 1,000 mg; Mn, 10,000 mg; Zn, 8,000 mg; I, 120 mg; Se, 25 mg; Co, 20.5 mg; Butylated hydroxy toluene (BHT), 12,500 mg; Beta-carotene, 400 mg; Canthaxanthin, 400 mg; [2] Analyzed; [3] Calculated; [4] Apparent metabolizable energy concentrations corrected to zero nitrogen balance (AME_N), calculated according to the energy estimation equation of the World's Poultry Association (Vogt, 1986).
