# Supplementary material for: Bone Health or Performance? Adaptation Response of Genetically Divergent Chicken Layer Lines to a Nutritive Calcium Depletion
Source: Animals (Basel). 2020 Sep 14;10(9):1645. doi: 10.3390/ani10091645 (PMC7552123; doi:10.3390/ani10091645)
Supplement: Supplementary file 1 [file animals-10-01645-s001.zip › Supplement_TableS3.pdf]

Supplementary Material

Table S3. Least squares means ± standard errors for egg quality traits during the last three consecutive days of preliminary (Pre), first (R1) and second recovery period (R2) in control (CON) and calcium deficient (DEF) groups of four layer lines (WLA, R11, BLA, L68).

	WLA		R11		BLA		L68	
**Period**	**CON**	**DEF**	**CON**	**DEF**	**CON**	**DEF**	**CON**	**DEF**
**Egg weight (g)**								
Pre	59.16 ± 0.87	58.19 ± 0.62	46.13 ± 0.42	45.91 ± 0.34	58.83 ± 0.86	59.83 ± 0.59	47.72 ± 0.69	47.62 ± 0.45
R1	60.12 ± 0.78	60.65 ± 0.5	49.34 ± 0.41	49.18 ± 0.3	61.57 ± 1.23	60.36 ± 0.83	52.68 ± 0.79	51.47 ± 0.49
R2	59.43 ± 0.8	53.68 ± 3.23	51.79 ± 1.0	52.48 ± 0.72	62.30 ± 2.45	63.00 ± 1.72	51.79 ± 1.0	52.48 ± 0.72
**Eggshell weight (g)**								
Pre	6.65 ± 0.12	6.68 ± 0.08	4.63 ± 0.08	4.71 ± 0.06	6.20 ± 0.13	6.32 ± 0.09	4.77 ± 0.17	4.73 ± 0.12
R1	6.94 ± 0.16	7.08 ± 0.11	5.01 ± 0.07	5.28 ± 0.06	6.52 ± 0.14	6.63 ± 0.1	5.42 [a] ± 0.1	5.14 [b] ± 0.06
R2	7.11 ± 0.16	7.14 ± 0.11	5.40 ± 0.15	5.60 ± 0.11	6.61 ± 0.49	6.87 ± 0.35	5.40 ± 0.15	5.60 ± 0.11
**Eggshell thickness (mm)**								
Pre	0.39 ± 0.01	0.39 ± 0.01	0.32 ± 0.01	0.31 ± 0.01	0.36 ± 0.01	0.37 ± 0.01	0.32 ± 0.01	0.31 ± 0.01
R1	0.39 ± 0.01	0.37 ± 0.01	0.32 ± 0.01	0.32 ± 0.01	0.36 ± 0.01	0.36 ± 0.01	0.33 [a] ± 0.01	0.31 [b] ± 0.01
R2	0.38 ± 0.01	0.39 ± 0.01	0.33 ± 0.01	0.33 ± 0.01	0.36 ± 0.01	0.37 ± 0.01	0.33 ± 0.01	0.33 ± 0.01
**Eggshell breaking strength (N)**								
Pre	49.26 ± 1.89	50.70 ± 1.35	34.36 ± 1.3	34.60 ± 0.98	44.12 ± 1.72	45.95 ± 1.15	38.05 ± 2.67	37.29 ± 1.85
R1	42.96 ± 2.86	43.70 ± 1.91	28.70 ± 1.59	29.89 ± 1.26	38.33 ± 1.47	38.78 ± 0.99	30.28 ± 2.0	29.91 ± 1.26
R2	37.97 ± 2.41	38.14 ± 1.73	25.64 ± 3.18	27.39 ± 2.27	31.6 ± 2.67	34.00 ± 1.86	25.64 ± 3.18	27.39 ± 2.27

[a,b] Means with no common superscript differed significantly within each parameter, experimental period and layer line (Tukey's HSD-Test, $p < 0.05$).
