# Supplementary material for: Bone Health or Performance? Adaptation Response of Genetically Divergent Chicken Layer Lines to a Nutritive Calcium Depletion
Source: Animals (Basel). 2020 Sep 14;10(9):1645. doi: 10.3390/ani10091645 (PMC7552123; doi:10.3390/ani10091645)
Supplement: Supplementary file 1 [file animals-10-01645-s001.zip › Supplement_TableS4.pdf]

Supplementary Material

Table S4. Mean ($\bar{x}$) daily feed consumption (g) during periods of calcium depletion (D2, D3) and recovery (R1, R2) in control (CON) and calcium deficient (DEF) groups of combinations of two layer lines each.

	WLA / L68 combination		BLA / R11 combination	
**Period**	**CON**	**DEF**	**CON**	**DEF**
D1	115.50	113.20	121.80	101.20
R1	115.80	114.90	110.30	115.50
D2	104.30	104.40	110.70	101.00
R2	119.30	123.90	118.10	123.40
D3	117.20	112.10	121.20	107.90
